# Silanization-Modified Lignin Nanoparticles for Paper Coating with Enhanced Liquid and Vapor Barriers, Frication Resistance, and Self-Cleaning Properties

**DOI:** 10.3390/polym17152066

**Published:** 2025-07-29

**Authors:** Wen Chen, Ren’ai Li, Yunfeng Cao, Chunjie Ye, Zhulan Liu, Huining Xiao

**Affiliations:** 1 International Innovation Center for Forest Chemicals and Materials, Jiangsu Co-Innovation Center of Efficient Processing and Utilization of Forest Resources and Jiangsu Provincial Key Lab of Sustainable Pulp and Paper Technology and Biomass Materials, Nanjing Forestry University, Nanjing 210037, China; chenwen20000727@163.com (W.C.); lirenai@njfu.edu.cn (R.L.); yunfcao@163.com (Y.C.); 2 Ningbo Asia Paper & Fibre Co., Ltd., Ningbo 315000, China; yechunjie@mail.zhonghua-paper.com; 3 Department of Chemical Engineering, University of New Brunswick, Fredericton, NB E3B5A3, Canada

**Keywords:** lignin nano-particle, silanization modification, paper coating, barrier properties

## Abstract

Paper’s inherent hydrophilicity and porosity cause inadequate barrier properties, failing under high humidity/temperature. This study successfully developed a hydrophobic nanocoating agent (xLNPs-OTS) through silanization modification using D276 (lignin nanoparticles with a diameter of 276 nm) as the substrate and OTS (octadecyltrichlorosilane) as the functionalizing agent. By applying the coating to paper surfaces followed by a hot-pressing process, the paper achieved comprehensive performance enhancements, including superior water, oil, and vapor barrier properties, thermal stability, mechanical strength, frictional resistance, and self-cleaning capabilities. The Cobb 60 value of LOTSC3.5T120t30 (the coating made from the OTS silanized lignin with the coating amount of 3.5 g/m^2^ and a hot-pressing at 120 °C for 30 min) coated paper is as low as 3.75 g/m^2^, and can withstand hot water at 100 °C for 60 min. The Cobb 60 value of the LOTSC20T120t30 (the coating made from the OTS silanized lignin with the coating amount of 20 g/m^2^ and a hot-pressing at 120 °C for 30 min) coated paper is reduced to 0.9 g/m^2^, the Kit grade is 6, and all coated papers are endowed with self-cleaning features. This study advances lignin’s high-value utilization, driving sustainable packaging and supporting eco-friendly paper material development.

## 1. Introduction

The global annual production of plastic exceeds 400 million tons. However, only 8% of them are recycled, and 8 million tons enter water bodies annually. Packaging materials account for 30% of the total plastic output. With the development of e-commerce and logistics industries, disposable plastics are commonly used as courier packaging [1]. However, most of them are traditional petroleum-based plastics, which are difficult to degrade during landfilling, and the ocean dumping process, and produce a large amount of microplastics to damage the ecological environment and threaten human health through the food chain transmission [2]. Under the “Plastic Restriction Order” and the “Double Carbon” goal, the development of biodegradable alternatives has become a significant challenge for the international community [3,4].

Recently, paper-based materials have been taken as an alternative to plastic for food packaging and logistics due to their renewable nature, biodegradability, and cost-effectiveness [5]. However, their porous structure and hydrophilic cellulose/hemicellulose components (rich in hydroxyl groups) result in strong liquid absorption, which causes compromised mechanical strength and limited broader applications. As a result, it is essential to enhance the resistance of paper to water, oil or vapor urgently [6,7]. The current industrialized program mostly uses petroleum-based barrier agents, such as film-applied polyacrylate [8,9] or perfluorooctanoic acid (PFOA) [10]. They are effective but difficult to degrade or repulp, which can pollute the environment and impede paper recycling. These coatings fail to align with sustainability goals, creating a critical need for bio-based alternatives. Although some bio-based coating agents, including chitosan [11], starch [12], Zein, algin, and so on, are eco-friendly; their inherent hydrophilicity leads to moisture resistance failure in high-humidity environments [13,14]. Therefore, the development of bio-based coating systems that combine high barrier properties with environmental friendliness has become a central challenge in green materials research.

As the second largest natural renewable resource in terms of reserves (after cellulose), lignin is non-toxic, widely available and inexpensive, while the rigid crosslinked aromatic structure brings unique hydrophobicity advantages [15,16]. Its performance can be modulated by chemical modification because of the hydroxyl, carboxyl, and other reactive groups that exist in the molecule [17]. Lignin nanoparticles (LNPs) can be used as hydrophobic fillers to enhance paper barrier properties and build surface micro-nano-structures through particle size/form modulation to form nano-effects, showing potential for high-value applications in the development of green paper coating agents [18,19]. Lignin, as a renewable hydrophobic polymer, has been explored for paper coating, but prior efforts face critical limitations. A kind of fully bio-based lignin/cellulose paper with multiple barrier effects and excellent antimicrobial properties was prepared through a simple one-step hot pressing process [20]. The sustainable lignin-coated paper composite that is water and oil resistant was developed by implementing a continuous lignin micro and nanoparticle (LMNP) layer on the surface of cellulose paper [21]. However, the above studies required a large amount of coating, which even amounted to 100 g/m^2^. Therefore, its economic feasibility is limited. The long-chain octadecyl group (C18) in OTS significantly reduces surface energy, endowing superhydrophobicity. Wei et al. [22] prepared lignin-based functional coatings with superhydrophobicity using OTS-modified lignin, triphenol epoxy resin (PTEP), and zinc oxide nanoparticles (ZnO), but there was no mention of the barrier properties of the coatings against oils. Additionally, the use of non-renewable epoxy resin compromised biodegradability. Wang et al. [23] reported lignin-based waterborne polyurethane (LWPU) coatings with polydimethylsiloxane (PDMS) modification, which showed good water resistance but failed to address high-temperature stability (e.g., resistance to 100 °C hot water), limiting applications in packaging for hot liquids.

This modification system thus innovatively integrates the high reactivity of small LNPs, the dual-functional modification of OTS, and structural optimization via hot-pressing, enabling low-cost, high-performance coatings that outperform existing lignin-based or petroleum-derived alternatives. In order to further break through the performance bottleneck, according to the team’s previous research [24], small-scale LNPs with a high density of surface active sites (i.e., D276) were screened out, and through the directional grafting of octadecyltrichlorosilane (OTS), the silanized lignin nanoparticles (xLNPs-OTS) were prepared, which were dissolved in ethanol and then sprayed onto the surface of the paper substrate to construct a low surface energy barrier on the paper surface, then combined with the hot pressing process to realize the deep penetration and densification of coating and built a synergistic mechanism involving “hydrophobicity-densification-bonding” on the paper, which enhances barrier properties at high temperature, mechanical strength, and super hydrophobicity. Additionally, superhydrophobicity provides the paper with self-cleaning capabilities. This innovation offers a sustainable alternative to non-degradable plastic coatings for supporting the eco-friendly evolution of the packaging industry.

## 2. Materials and Methods

### 2.1. Materials

Black liquor from hardwood kraft pulping was provided by Asia Pulp & Paper Group (APP), Haikou, China. Sulfuric acid (98 wt.%) was purchased from Zhejiang Hanuo Chemical Co., Ltd., Hangzhou, China. Chemically pure castor oil, toluene, and n-heptane were all purchased from Nanjing Chemical Reagent Co., Ltd., Nanjing, China. Acetone, 1-methylimidazole and octadecyltrichlorosilane were purchased from Shanghai McLean Biochemical Co., Ltd., Shanghai, China. Chemically pure anhydrous ethanol was provided by Xilong Science Co., Ltd., Guangzhou, China. The commercial multifunctional copy paper (Deli Group, Ningbo, China, NO. 33129) was taken as the raw paper (U-p) with a grammage of 80.0 g/m^2^ and a thickness of 110 μm.

### 2.2. Preparation of Lignin Nanoparticles (LNPs)

According to the previous work, LNPs with a small size contain numerous active hydroxyl groups, which is beneficial for chemical modification. Here, small-sized LNPs were selected as the starting material to synthesize hydrophobic xLNPs-OTS via silylation. An appropriate amount of black liquor was stirred at 30 °C at 150 r/min continuously, while gradually dropwise introducing a 50% (*w*/*w*) sulfuric acid solution till the solution was adjusted to pH2, and the system was kept stirring for an additional 30 min at the same temperature before standing for 12 h. The resulting LNPs were separated via centrifugation, washed with distilled water until neutral and freeze-dried to obtain small-scale LNPs with a diameter of 276 nm.

### 2.3. Preparation of Silanized Lignin Nanoparticles (xLNPs-OTS)

**Figure 1:**
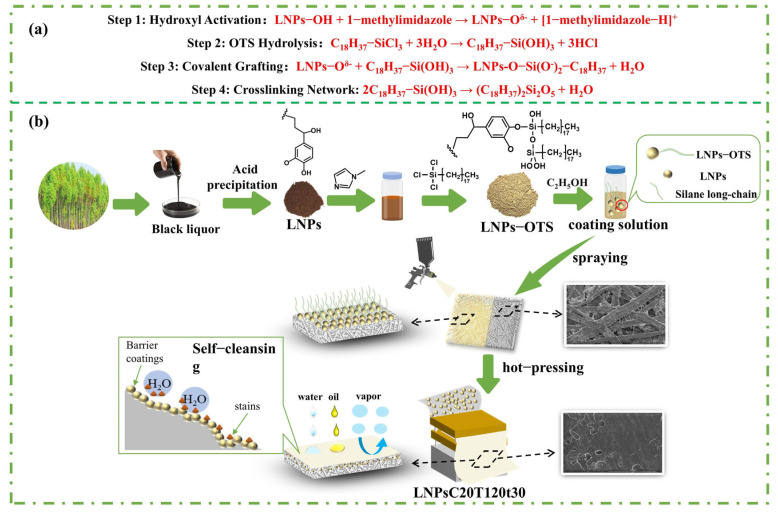
(**a**) LNPs-OTS silanization reaction mechanism; (**b**) Preparation process of LNPs-OTS surface coating agents and paper surface application mechanism.

A total of 4 g of LNPs and 36 g of deionized water were mixed in a four-necked flask equipped with a mechanical stirrer, pressure-equalizing funnel, thermometer, and reflux condenser. Then, 1-methylimidazole was added for activation, and the mixture was stirred at room temperature for 1 h. A total of 5 g of acetone and 5 g of octadecyltrichlorosilane (OTS) were added dropwise into the flask separately through the pressure-equalizing funnel. The reaction was maintained at 50 °C with constant stirring for 24 h to allow covalent grafting of OTS onto hydroxyl groups on the LNP surface. Afterward, the mixture was centrifuged to collect the precipitate, which was ultrasonically washed with anhydrous ethanol to remove physically adsorbed OTS (Figure 1a). The final product was vacuum-dried at 40 °C for 24 h to yield light-brown silanized lignin nanoparticles (LNPs-OTS) in powder form.

The influence of OTS content on the water resistance, vapor barrier properties, mechanical strength, and hydrophobicity of paper treated with xLNPs-OTS coating was systematically investigated through individual experiments. The detailed synthesis mechanism and formulation for LNPs-OTS are shown in Table 1 and Figure 1, respectively.

### 2.4. Surface Coating

The coating solution was prepared by dissolving LNPs or modified xLNPs-OTS in anhydrous ethanol, followed by ultrasonic dispersion to obtain a homogeneous 2 wt% suspension. U-p was then surface-coated using an air-assisted spray gun at room temperature (single-layer coating). The suspension was uniformly coated onto U-p substrates and air-dried until complete ethanol evaporation. To improve adhesion between the coating and substrate, the coated paper sheets were hot-pressed with a flat-plate vulcanizing press under 10 MPa pressure. The temperature and duration parameters were optimized as shown in Figure 1. Subsequently, all these coated papers were conditioned at 23 °C and 50% relative humidity (RH) for at least 24 h to equilibrate the moisture content for further analysis.

Table 2 was designed based on a single-factor experimental strategy, aiming to systematically investigate the independent effects of four key variables on coated paper performance: (1) OTS dosage during silanization (reflected by xLNPs-OTS types: LNPs-OTS, 2LNPs-OTS, 3LNPs-OTS, corresponding to Table 1’s molar ratios of -OH:OTS = 1:1, 2:1, 3:1); (2) coating amount of xLNPs-OTS; (3) hot-pressing temperature; and (4) hot-pressing duration. The formulations were designed to isolate each variable while keeping others constant, ensuring clear attribution of performance changes to specific factors.

### 2.5. Characterization of xLNPs-OTS

Fourier transform infrared spectroscopy (FT-IR) detection was performed on a VERTEX 80 V FTIR spectrometer (Bruker, Billerica, MA, USA) using an ATR accessory. Each spectrum was obtained by averaging 32 scans in the frequency range from 4000 to 400 cm^−1^ with a spectral resolution of 4 cm^−1^.

The surface elemental composition of xLNPs-OTS was characterized by X-ray photoelectron spectroscopy (XPS) using a AXIS UltraDLD spectrometer (Shimadzu, Kyoto, Japan). All spectra were acquired under ultra-high vacuum conditions across a binding energy range of 0–1200 eV. High-resolution scans of C1 s, O1 s, and Si 2p core-level spectra were subsequently performed to analyze chemical states.

The particle size and zeta potential of LNPs were measured before and after modification using a nanoparticle size laser (Zetasizer Nano-Zs, Malvern, Worcestershire, UK). The surface morphology of the samples was observed using a scanning electron microscope system (Quanta 200, FEI, Hillsboro, OR, USA). The thermal stability of the samples was measured using a thermogravimetric analyzer (TGA209 F1, NETZSCH, Bavaria, Germany).

### 2.6. Paper Characterization

The water resistance of the paper samples was characterized by determining the Cobb 60 value according to the TAPPI T441 method. Using a Cobb tester (ZBK-100, Yuanming Compact Tester, Jilin, China), the paper samples were contacted with 100 mL distilled water or 100 mL of hot water at 100 °C for 60 s, respectively, then wiped with absorbent paper, and the Cobb 60 value and the 100 °C Cobb60 value of the paper samples were finally obtained by dividing the weight difference between the paper samples before and after contact with water by the area of the paper samples. All the tests were conducted in triplicate to calculate the final average value.

The Kit rate was taken to represent the oil resistance of paper samples according to the standard of TAPPI T559. The standard oil solution with different proportions of castor oil/n-heptane/toluene (rated from 1 to 12) for Kit rating was dropped on the surface of the paper from a height of 1 cm, then removed with a tissue after 15 s, and spots on the surface of the pattern were visually observed. The maximum-rated oil solution that remained on the paper surface without causing staining was reported as the Kit rating. At least five specimens of each sample were tested to calculate the mean value.

The tensile index and elongation at break were measured by a horizontal tensile testing machine (GB-KYZB, GBPI, Guangzhou, China) according to the reference standard TAPPI T494.

The water vapor transmission rate (WVTR) of paper was evaluated by using the water transmission rate measurement system (W3-031, MOCON, Brooklyn Park, MN, USA) according to the reference standard GB/T 1037-2021.

A dynamic contact angle analyzer (T200-Auto 3 plus, Biolin Scientific AB, Gothenburg, Sweden) was used to further investigate the hydrophobic ability of paper materials in contact with water. Approximately 4 μL of distilled water was dropped on the paper sample surface, and the resulting contact angle was recorded for 15 s and 300 s.

The surface and cross-sectional micromorphology of paper were observed using a SEM system (Quanta 200, FEI, OR, USA). A thermogravimetric analyzer (TGA209 F1, NETZSCH, Seelbach, Germany) was used to measure the thermal stability of paper.

The stability of the coating was assessed by placing the test paper flat on the sandpaper with the coating facing downwards, adding a 100 g weight on the paper and sliding it across the sandpaper for 50 repetitions to observe the changes on the paper surface, and measure the contact angle post-rubbing [25]. The self-cleaning capability of the coated paper was shown by attaching the paper onto a slide, then applying drops of staining solution and floating ash on one end of the paper, followed by water drops to monitor surface changes.

## 3. Results and Discussion

### 3.1. Properties of xLNPs-OTS

**Figure 2:**
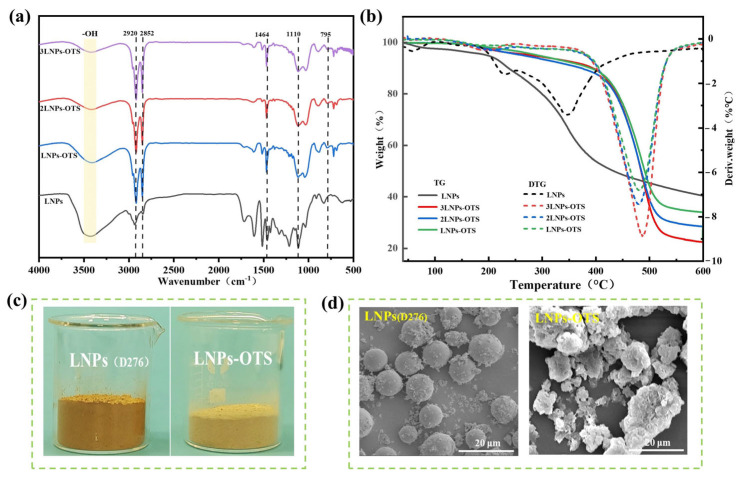
Characterization of LNPs and xLNPs-OTS: (**a**) IR; (**b**) Thermal stability; (**c**) Optical images; (**d**) SEM.

Small-scale LNPs were prepared following the previous protocol [24]. Their particle size was measured to be 276 nm using a nanoparticle size laser, and the hydroxyl group (-OH) content in LNPs was quantified at 9.65 mmol/g via acetylation hydrolysis [26]. Hydrophobic nanomaterials (xLNPs-OTS) were successfully synthesized through silanization modification using LNPs (D276) as the base and octadecyltrichlorosilane (OTS) as the functionalization reagent. The successful silanization of lignin was confirmed using multiple characterization methods. The chemical structure changes of LNPs before and after modification were revealed using FT-IR. As shown in Figure 2a, the intensity of the O-H stretching vibration peak at 3470 cm^−1^ of the modified xLNPs-OTS was significantly decreased, which confirms that the silane chains successfully replaced the hydroxyl groups on the surface of LNPs [22]. Enhanced C-H stretching vibrations at 2920 cm^−1^ (asymmetric) and 2852 cm^−1^ (symmetric) indicated alkyl chain grafting. xLNPs-OTS was successfully synthesized with long or branched Si-O-Si groups at 1110 cm^−1^, and the Si-O-Si absorption became broader and more intricate as the siloxane chain became longer or branched. The widened Si-O-Si absorption band at 1110 cm^−1^ demonstrated extended/polymerized siloxane chains [27,28,29]. LNPs started to decompose at lower temperatures, and the pyrolysis time of xLNPs-OTS was much later than that of LNPs as the stronger thermal stability of silane chains than hydroxyl groups. The thermal stability of modified xLNPs-OTS was enhanced compared with LNPs, and the mass residue at 600 °C was approximately 32.78%, which indicated that the modified LNPs had good thermal stability (Figure 2b) [30]. The effect of silanization modification on the micro-morphology of LNPs was analyzed using scanning electron microscopy (SEM), as shown in Figure 2d. The raw LNPs without modification exhibited a uniform spherical morphology with smooth surfaces and narrow size distribution, which is consistent with the homogeneous nucleation mechanism governed by π-π stacking during acid precipitation. In contrast, xLNPs-OTS exhibited notably rougher surfaces with porous structures and irregular protrusions. This morphological alteration could be attributed to the covalent grafting of OTS octadecyl chains onto LNP surfaces via Si-O-C bonding, which disrupts the original π-π stacking order. This led to molecular chain rearrangement and steric hindrance effects from long alkyl chains on OTS, promoting the formation of branched surface protrusions [22,31]. The zeta potential of modified xLNPs-OTS shifted from −28.1 mV to +1.1 mV, indicating weakened electrostatic repulsion and enhanced van der Waals forces, resulting in closely packed aggregates with a increased size (1523 nm). As can be observed from Figure 2c, the LNPs-OTS was more loosely packed and had a light-yellow color, and the successful silylation altered the chromophores of the LNPs and introduced the long-chained silanes for modification [32].

**Figure 3:**
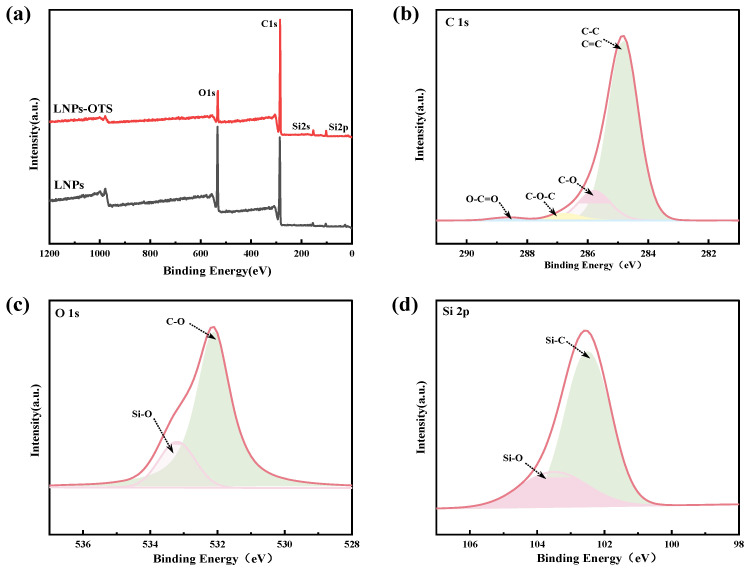
(**a**) XPS full spectrum of LNPs and LNPs-OTS; LNPs-OTS (**b**) C 1s spectrum; (**c**) O 1s spectrum; (**d**) Si 2p spectrum.

X-ray photoelectron spectroscopy (XPS) was employed to analyze LNPs and LNPs-OTS to examine their chemical composition. As shown in Figure 3, LNPs exhibited dominant characteristic peaks at C 1s (284 eV) and O 1s (532 eV), consistent with the elemental stoichiometry of lignin (C_9_H_10_O_2_). The modified LNPs-OTS exhibited additional characteristic peaks for Si 2p and Si 2s, which confirmed that the silane moieties were successfully grafted onto LNPs. Two peaks were fitted to the binding energy curves of Si 2p peaks in LNPs-OTS spectra [33]. The Si-C bond at 102.5 eV corresponds to the covalent bonding between the LNP silicon atom and the aromatic ring. This peak originates from the covalent linkage between the silicon atom in OTS and the carbon atom in LNPs via an oxygen bridge (i.e., Si-O-C bonding). This is direct evidence of OTS grafting onto the hydroxyl groups (-OH) of LNPs, as the trichlorosilane group of OTS undergoes condensation with -OH groups on LNPs, forming stable Si-O-C covalent bonds. A peak at 103.5 eV, attributed to Si-O bonding, which originates from the self-condensation of silanol groups (-Si(OH)_3_) formed by hydrolysis of OTS, leading to the formation of a Si-O-Si cross-linked network [34]. The above results indicate that nanoscale LNPs provide abundant grafting sites and OTS is successfully grafted onto LNPs, which results in a surface rich in long alkyl chains. These confirm directly demonstrate the introduction of Si-containing groups via chemical bonding, not physical adsorption. This modification could effectively reduce the surface energy and further enhance the hydrophobicity of the LNPs.

### 3.2. Water Resistance of Coated Paper

**Figure 4:**
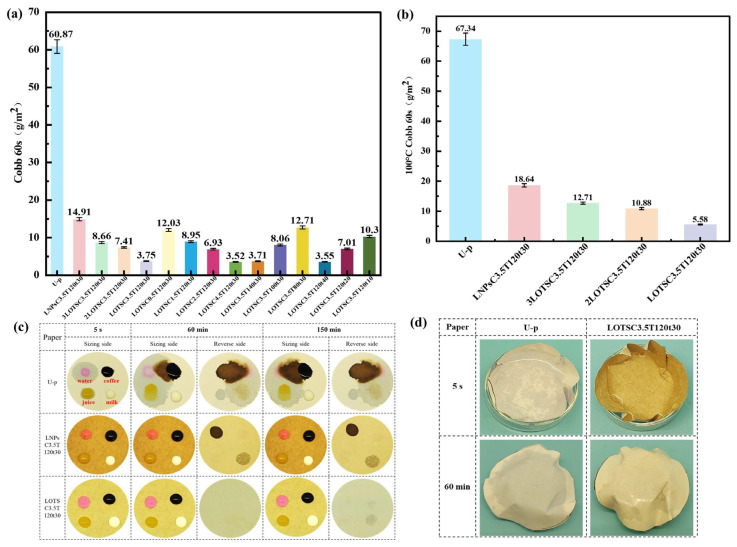
(**a**) Impact of OTS dosage, xLNPs-OTS coating, and thermal pressing parameters (temperature/time) on coated paper waterproofing during silanization; (**b**) 100 °C hot water Cobb 60 of xLNPs-OTS coated paper; (**c**) barrier performance of U-p, LNPsC3.5T120t30, and LOTSC3.5T120t30 against water, coffee, juice, and milk; (**d**) liquid resistance of U-p and LOTSC3.5T120t30 containers containing 100 °C hot water.

Next, the effects of OTS dosage during silanization, xLNPs-OTS coating amount, hot-pressing temperature, and hot-pressing duration on the properties of xLNPs-OTS coating paper were investigated. The water resistance of paper was evaluated through Cobb 60 value measurements. As shown in Figure 4a, the untreated base paper (U-p) exhibited a high Cobb 60 value of 60.87 g/m^2^ due to its porous structure and hydrophilic -OH groups on the fibers. Surface coating with raw LNPs (LNPsC3.5T120t30) without modification reduced the Cobb 60 value to 14.91 g/m^2^. Remarkably, the LNPs-OTS coating treatment (LOTSC3.5T120t30) achieved a drastic reduction in Cobb 60 value to 3.75 g/m^2^, representing a 93.8% decrement compared with U-p. This superior performance demonstrates that the hydrophobic silanized xLNPs-OTS significantly outperforms unmodified LNPs in water barrier performance. This is attributed to the inherent hydrophobicity of LNPs and their melt-flow behavior during hot pressing, leading to effective infiltration of interfiber pores and densification of the paper matrix [35,36]. The C18 alkyl chains on the surface of the xLNPs-OTS formed a low-surface-energy barrier, and the Si-O-Si network formed a continuous phase, which further enhanced the water resistance of paper after xLNPs-OTS coating [37,38]. With the decrease of OTS dosage during silanization, the water resistance of xLNPs-OTS coated paper showed a decreasing trend, but still maintained at a high level. As can be seen in Figure 4b, the xLNPs-OTS-coated paper has the same excellent barrier ability against hot water at 100 °C. The LOTSC3.5T120t30 sample exhibited a 100 °C Cobb 60 value of 5.58 g/m^2^, which is 91.3% lower than that of U-p. It can significantly broaden their potential applications in high-temperature liquid packaging scenarios.

In Figure 4a, under optimized hot-pressing conditions (120 °C, 30 min), the water resistance of LNPs-OTS-coated paper represented a dosage-dependent enhancement. The Cobb 60 value decreased gradually from 3.75 g/m^2^ to 3.52 g/m^2^ as the coating amount increased from 3.5 g/m^2^ to 4.5 g/m^2^, showing only a 6% improvement at the higher dosage. This slight enhancement indicates that the 3.5 g/m^2^ dosage has already achieved nearly optimal pore sealing. The influence of hot-pressing conditions on the water resistance of paper coated with LNPs-OTS (3.5 g/m^2^ dosage) was systematically investigated. As shown in Figure 4a, the Cobb 60 value exhibited a progressive reduction with rising hot-pressing temperature and duration. This improvement is attributed to enhanced melt penetration at higher temperatures, which improves the ductility of LNPs-OTS for better spreading and coverage on the paper surface. The thermally activated LNPs-OTS exhibited deep penetration between fibers, effectively sealing the hierarchical pore structure and thereby enhancing barrier performance. Overall, with an LNPs-OTS coating dosage of 3.5 g/m^2^ and hot-pressing at 120 °C for 30 min, the paper products could be obtained with excellent waterproofing and cost-effectiveness.

In Figure 4c, there are the barrier properties of the coated paper to different liquids, including water, coffee, juice, and milk. These four liquid drops were immediately absorbed and rapidly penetrated to the reverse side of U-p without any coating, which indicated that the U-p had poor barrier performance to liquids. On the modified LNPs coating paper (LNPsC3.5T120t30), the polar liquids (water, coffee, and juice) started to penetrate in trace amounts after 60 min, and the milk penetration was delayed to 150 min due to the hydrophobic nature. In contrast, the LOTSC3.5T120t30 sample maintained stable droplet morphology for over 150 min without backside penetration, which demonstrates superior barrier efficiency. The long-chain alkyl groups of LNPs-OTS formed a low-surface-energy barrier to inhibit the wetting of polar liquids, and the nanoscale protrusions on the surface coating increased the surface roughness and slowed the permeation of liquids. The liquid resistance of paper containers was evaluated under harsh thermal conditions using 100 °C hot water. As shown in Figure 4d, U-p containers exhibited complete hot water penetration rapidly within 5 s, which is due to accelerated liquid diffusion through thermally swollen cellulose fibers. In contrast, LOTSC3.5T120t30 containers exhibited remarkable thermal liquid resistance, with initial penetration observed after 60 min of continuous exposure. This enhanced resistance is due to the formation of a mechanical interlocking structure with the fibers by the Si-O-C covalent bonding, reinforced chemical bonding, and reduced coating porosity from 120 °C hot-pressing, resulting in denser paper fibers and improving the liquid resistance of LNPs-OTS coated paper. Compared with previous studies [24], when LNPs-OTS were used for surface sizing of paper after hot-pressing, a much lower amount of sizing (3.5 g/m^2^, compared to 20 g/m^2^ previously) can achieve excellent water resistance (Cobb60 3.75 g/m^2^, compared to 13.86 g/m^2^ before), and also possess excellent barrier properties against hot water. Hot-pressing promotes melt penetration into fiber pores, forming a compact barrier that outperforms the loose structures reported in prior studies.

### 3.3. Micromorphology of Coated Paper

**Figure 5:**
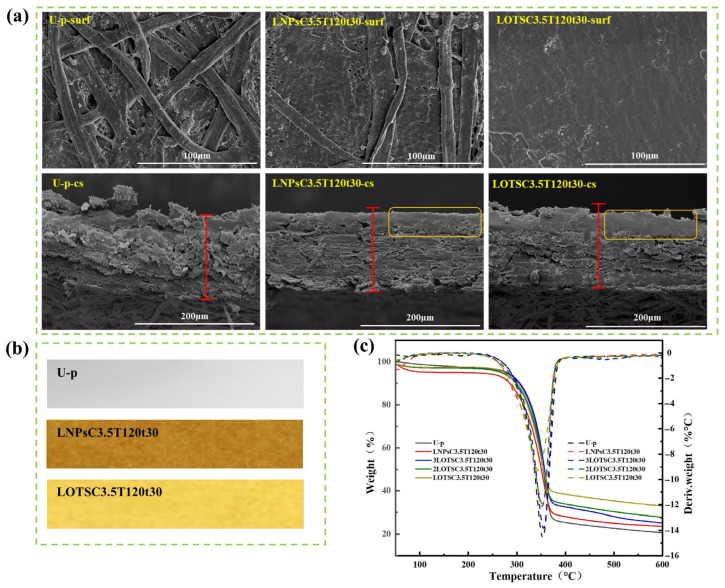
U-p, LNPsC3.5T120t30 and LOTSC3.5T120t30 (**a**) SEM images: upper is the paper surface, the down is the paper cross-section; (**b**) optical images; (**c**) TG and DTG.

The microstructural evolution of paper surfaces before and after coating treatment was analyzed via scanning electron microscopy (SEM), as shown in Figure 5a. U-p exhibited an open network structure composed of intertwined hydrophilic cellulose fibers with high porosity, which results in poor barrier properties [39]. After coating with LNPs or xLNPs-OTS, a continuous coating layer uniformly covered the fiber surfaces and effectively blocked liquid penetration pathways. During hot-pressing, molten LNPs or xLNPs-OTS penetrated into inter-fiber spaces, achieving superior pore-filling efficiency, thereby enhancing the barrier performance. Cross-sectional analysis showed increased paper matrix densification after hot-pressing, resulting in compressed fiber–fiber interfaces forming a more compacted structure. As observed in Figure 5b, the LOTSC3.5T120t30 sample displayed a lighter color compared with LNPsC3.5T120t30. Furthermore, compared with the previous reference [24], the color change is more pronounced (from dark brown to light yellow). It is attributed to the disruption of chromophore groups in LNPs during silanization, while broadening its application potential in upscale packaging sectors requiring visual uniformity.

### 3.4. Thermal Stability of Coated Paper

The thermal stability of U-p, LNPsC3.5T120t30, and xLOTSC3.5T120t30 papers was characterized, and the results are shown in Figure 5c. All these coated papers have the same amount of coating and less gluing, and all of them exhibit similar thermodynamic behavior. An initial weight loss stage between 50–120 °C was attributed to moisture evaporation. A major thermal decomposition stage was observed from 250 to 360 °C, characterized by rapid mass loss corresponding to cellulose degradation and coating decomposition. The silanized LNPs-OTS coating agent significantly improves the thermal stability of the paper through chemical bonding; in particular, 2LOTSC3.5T120t30 or 3LOTSC3.5T120t30 coated paper is more thermally stable than LNPsC3.5T120t30. Overall, all these coated papers exhibit good thermal stability and can withstand high temperatures above 200 °C for everyday food packaging applications, such as oven or microwave heating.

### 3.5. Mechanical Strength of Coated Paper

**Figure 6:**
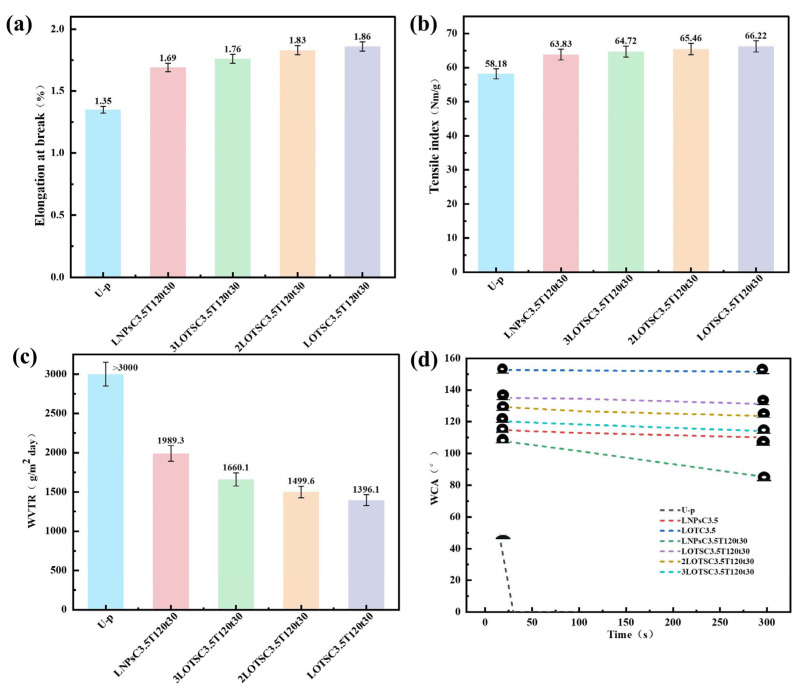
U-p, LNPsC3.5T120t30, and LOTSC3.5T120t30. (**a**) Elongation at break. (**b**) Tensile index; (**c**) WVTR; (**d**) WCA.

The tensile index and elongation at break were measured to evaluate the mechanical strength of the paper. As shown in Figure 6a,b, U-p exhibited the weakest mechanical performance with an elongation at break of 1.36% and a tensile index of 58.18 N·m/g. After coating with LNPs or xLNPs-OTS, the mechanical strength of the paper was significantly enhanced. The LOTSC3.5T120t30 sample exhibited an elongation at break of 1.86% and a tensile index of 66.22 N·m/g. After hot-pressing, LNPs and xLNPs-OTS filled the pores between the interweaved fibers to enhance the stress transfer efficiency, densify the overall structure of the paper, increase the fiber interactions and friction, and improve the mechanical strength subsequently [21]. With the increment of OTS addition during silanized modification, the mechanical strength of paper after xLNPs-OTS coating was improved. It might be attributed to the fact that the surface of LNPs became rougher after OTS modification, the friction between LNPs and paper fibers increased, the retention ability in the fiber pores improved, the interactions between fibers increased, and the mechanical properties of paper finally enhanced.

### 3.6. Vapor Resistance of Coated Paper

With the development of paper-based products, more functions are expected from paper-based materials. In the field of food packaging, where it is important to maintain the quality and freshness of food, moisture may lead to food spoilage, which requires paper-based packaging materials to have the ability to block vapor. WVTR in Figure 6c serves as a test standard for evaluating the barrier properties of paper to water vapor. The surface of the raw paper U-p is rough and porous, and its barrier to water vapor is too weak, as it exceeds the test range of the machine (WVTR > 3000 g/m^2^·day). In contrast, the WVTR of xLOTSC3.5T120t30 coated paper exhibited a progressive reduction with increasing OTS content in xLNPs-OTS. Hot-pressing treatment enhanced the uniformity and density of the xLNPs-OTS coating on the paper surface, while concurrently compacting the internal fiber matrix and reducing inter-fiber porosity. These structural modifications collectively improved moisture barrier performance, with the optimized LOTSC3.5T120t30 sample achieving a WVTR of 1396.1 g/m^2^·day, representing a 50.3% reduction compared to U-p. This technological advancement significantly broadens the potential applications of hot-pressed paper in moisture-resistant packaging, particularly for humidity-sensitive product storage and transportation.

### 3.7. Hydrophobicity of Coated Paper

**Figure 7:**
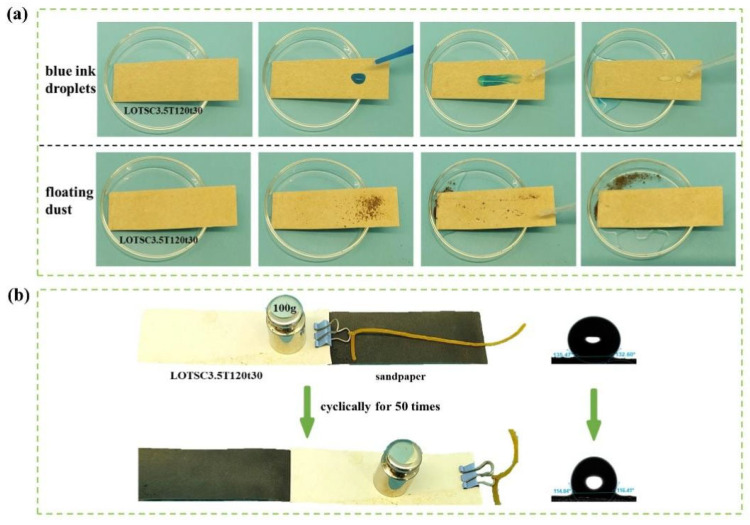
LOTSC3.5T120t30. (**a**) Self-cleaning. (**b**) Friction resistance.

Water contact angle (WCA) was systematically performed to evaluate the hydrophobicity of packaging materials. As shown in Figure 6d, the U-p sample exhibited a WCA of around 50°, showing strong liquid affinity and hydrophilic properties with complete droplet absorption (WCA = 0°) within 30 s. When LNPs are coated on the paper surface but not hot-pressed, the surface WCA is greater than 110°, whereas when LNPs-OTS is coated without hot-pressing, the surface achieves superhydrophobicity with a WCA of greater than 150°, but the poor retention of the coatings restricts practical application. The LNPsC3.5T120t30 sample maintained hydrophobicity with a WCA of 90° after 300 s, indicating enhanced water resistance from LNPs. Increasing OTS content in xLOTSC3.5T120t30 significantly improved surface hydrophobicity, with the optimized LOTSC3.5T120t30 sample maintaining a WCA above 135° initially and around 130° after 300 s, which could be attributed to the successful grafting of OTS chains onto LNPs. This modification reduced surface energy through hydroxyl group substitution by OTS chains, decreased intermolecular attraction, and increased surface roughness, collectively enhancing hydrophobicity. After hot-pressing, the uniformly sprayed xLNPs-OTS formed a dense barrier layer, converting hydrophilic paper into a hydrophobic substrate with self-cleaning capabilities. Compared with the previous studies, the water contact angle of the coated paper after 300 s increased by twice (130°, compared with 65° before). This long-term hydrophobic stability arises from covalently grafted OTS long alkyl chains (reducing surface energy) and hot-pressing-induced densification (enhancing coating adhesion). In Figure 7a, blue ink droplets and floating dust on the LOTSC3.5T120t30 surface were completely removed through minimal water rinsing, leaving no residue and achieving a dry surface, thereby validating its autonomous contamination resistance.

### 3.8. Friction Resistance of Coated Paper

The coated paper LOTSC3.5T120t30 was placed flat on sandpaper with the coating facing downward. A 100 g weight was then applied to the paper, and it was cyclically dragged and rubbed on the sandpaper for 50 repetitions to observe the changes and measure the contact angle of the paper surface after friction. As shown in Figure 7b, the WCA after friction remained above 110°, representing only a 12% reduction from the initial value (135°). This slight reduction confirms the strong interfacial adhesion of hot-pressed LNPs-OTS coatings, which preserved their hydrophobic functionality under mechanical stress [40]. The enduring hydrophobicity and intact coating structure highlight enhanced durability, significantly broadening the potential applications of these modified papers in high-wear scenarios such as reusable packaging and industrial-grade barrier materials.

### 3.9. Oil Resistance of Coated Paper

**Figure 8:**
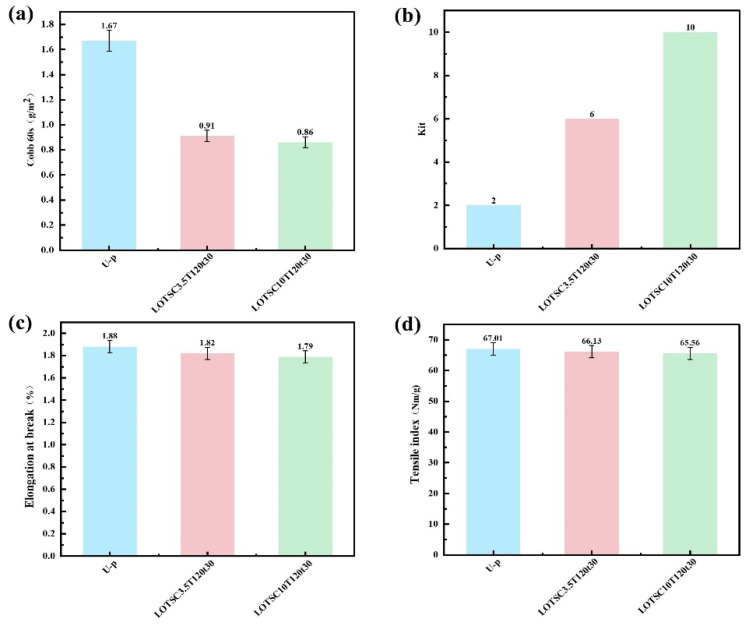
Characterization of coated paper with high coating amount. (**a**) Water resistance. (**b**) Kit rate. (**c**) Elongation at break. (**d**) Tensile index.

From the above research, it can be seen that a lower coating amount could help to enhance the waterproof, vapor resistance, thermal stability, and mechanical strength properties of coated paper, but the oil resistance of the coated paper is compromised, even with a coating amount of 3.5 g/m^2^, resulting in Kit grade 0. This might be due to the thinness of the coating with a relatively low coating amount, resulting in a dense layer formed through hot-pressing that is insufficient to block the oil molecules diffusion effectively [20]. As shown in Figure 8a, the water and oil resistance of coated paper are progressively improved as the LNPs-OTS coating amount increases to 10, 20, or even 30 g/m^2^. If the coating amount rose to 30 g/m^2^, the Cobb 60 value decreased to 0.86 g/m^2^, and the grease barrier Kit rate reached 10, which indicated the formation of a complete and closed coating network with a higher coating amount. This compact structure prevented fiber exposure issues and effectively blocked lipid molecule diffusion pathways, thereby enhancing oil resistance. Even though there is a negligible decrease in mechanical strength, including elongation at break and tensile index, these might be attributed to the fact that the thicker coating layer raised overall brittleness and decreased flexibility, leading to decreased elongation at break [41,42]. Additionally, the thick coating exhibited susceptibility to stress-induced cracking and compromised tensile strength. Comprehensive evaluation determined 20 g/m^2^ as the optimal coating amount. The coated paper LOSTC20T120t30 achieved a Cobb 60 value of 0.91 g/m^2^ and a Kit rate of 6, which represented excellent both water and oil resistance to meet performance requirements for most practical applications while maintaining acceptable mechanical strength.

## 4. Conclusions

This study successfully developed a hydrophobic nanoscale coating agent (xLNPs-OTS) to improve the combination performance of paper materials, including water, oil, and vapor barriers, thermal stability, mechanical strength, friction resistance and self-cleaning properties, through silanization modification using D276 nm as the matrix and OTS as the functionalization reagent. The results demonstrated that nano-scaled LNPs enabled OTS covalent grafting on high-density reactive sites, where surface -OH groups were replaced by OTS with C18 alkyl chains. This modification decreased surface energy, increased roughness, and endowed exceptional superhydrophobicity to the paper with a WCA of 150° even after 300 s. If combined with hot-pressing, xLNPs-OTS melt penetrated between the interweaved fiber and fiber layers to form a synergistic multi-stage “hydrophobic-dense-bonding” barrier. The resulting coated paper LOTSC3.5T120t30 achieved a significant decrease in Cobb 60 value to 3.75 g/m^2^ with a 93.8% reduction from U-p and a 100 °C hot water Cobb 60 value of 5.58 g/m^2^, along with improved mechanical strength and self-cleaning capability. Optimization of the coating amount to 20 g/m^2^ further reduced the Cobb 60 value to 0.91 g/m^2^ and elevated the oil barrier to Kit rate 6, which could meet most application requirements. Moreover, a higher coating amount (30 g/m^2^) could improve oil resistance to Kit rate 10, even leading to a slight reduction in mechanical strength originating from increased coating thickness and brittleness. This work provides an innovative and sustainable solution for high-performance paper-based materials by integrating efficient barrier properties, mechanical strength reinforcement, and thermal stability. In the future, it will be possible to further explore the use of bio-based silanes as an alternative to OTS, thereby reducing the potential environmental risks associated with chlorinated silanes. Moreover, it is essential to continue to expand the multi-functional integration of the coating paper, such as antibacterial activity or UV shielding properties, to broaden its application for food packaging.

## Figures and Tables

**Table 1:** Parameters for xLNPs-OTS preparation.

Sample	LNPs (g)	OTS (g)	Molar Ratio of LNPs Hydroxyl to OTS n (-OH):n (OTS)
LNPs	4	-	-
LNPs-OTS	4	5	1:1
2LNPs-OTS	4	2.5	2:1
3LNPs-OTS	4	1.67	3:1

Annotation: “LNPs”-Unmodified lignin nanoparticles (control, no OTS); “LNPs-OTS”, “2LNPs-OTS”, “3LNPs-OTS”-Silanized LNPs with -OH:OTS molar ratios of 1:1, 2:1, 3:1.

**Table 2:** Parameters for paper coating.

Coating Sample ^a^ (LOTSCxTytz)	Lignin	Coating Amount (g/m^2^)	Hot-Pressing Temperature (°C)	Hot-Pressing Duration (min)
U-p	-	-	-	-
LNPsC3.5	LNPs			
LOTSC3.5	LNPs-OTS	3.5		
LNPsC3.5T120t30	LNPs		120	30
LOTSC3.5T120t30	LNPs-OTS			
2LOTSC3.5T120t30	2LNPs-OTS			
3LOTSC3.5T120t30	3LNPs-OTS			
LOTSC0.5T120t30	LNPs-OTS	0.5		
LOTSC1.5T120t30		1.5		
LOTSC2.5T120t30		2.5		
LOTSC4.5T120t30		4.5		
LOTSC10T120t30		10		
LOTSC20T120t30		20		
LOTSC30T120t30		30		
LOTSC3.5T140t30		3.5	140	
LOTSC3.5T100t30			100	
LOTSC3.5T80t30			80	
LOTSC3.5T120t40			120	40
LOTSC3.5T120t20				20
LOTSC3.5T120t10				10

^a^: “U-p”: unglued paper; “LOTSCxTytz”: OST—OTS dosage during silanization (reflected by xLNPs-OTS types: LNPs-OTS, 2LNPs-OTS, 3LNPs-OTS, corresponding to Table 1’s molar ratios of -OH:OTS = 1:1, 2:1, 3:1), Cx—coating amount of xLNPs-OTS, Ty—hot-pressing temperature, tz—hot-pressing duration.

## Data Availability

Data are contained within the article.
